# The Book World of Medicine and Science

**Published:** 1902-02-01

**Authors:** 


### The Book World of Medicine and Science.

An Experimental and Clinical Research into certain Problems relating to Surgical Operations. By George W. Crile, A.M., M.D., PhD., Professor of Clinical Surgery, Medical Department, Reserve University; Surgeon to St. Alexis Hospital; Associate Surgeon to Lakeside Hospital, Cleveland. (Philadelphia: J. B. Lippincott & Co. 1901. Pp. 196, with 24 figures. Price 12s. 6d.).

This work is the essay awarded the Alvarenga Prize for 1901 by the College of Physicians of Philadelphia, and while the greatest praise must be given for the painstaking care with which the various experiments on animals were carried out, it must be admitted that the practical value of the results gained, varies greatly. The first series of experiments described relate to operations on the vagus nerve. It is well shown how severely this nerve may be dealt with before any marked effect is produced on the animal, and that when both vagi are divided the respiratory mechanism is much more affected than the circulatory, all of which has been worked out often before. Several experiments were next undertaken to discover the effect of intravenous infusion of saline solution. The experiments on the physiologic actions of cocain and eucain when injected into tissues are, however, of far greater clinical importance. Indeed, if the methods of Dr. Crile were ever generally adopted they would go far to revolutionise the practice of operating and remove entirely the unpleasantness and danger attending general anæsthesia. It is conclusively proved that the injection of these substances into the spinal cord or a nerve trunk produces a complete "block," so that neither afferent nor efferent impulses can pass, "the conductivity being as completely interrupted as if the nerve were divided." Such a mode of procedure, before operating, might have great advantages over a general anæsthetic, for there is no fear of shock following even the most severe operations. Dr. Crile also demonstrates that arteries may be safely temporarily closed by the application of suitable forceps without injuring their walls. Several severe operations are described as having been successfully performed by these methods, such as amputation of the arm at the shoulder joint, amputation of the arm at the middle, and amputation of half of the "shoulder girdle" after the parts had been rendered anæsthetic by exposing—under the influence of cocaine—the brachial plexus and injecting minute quantities of that drug into the nerve trunks. In fact the total amount used in the operation first described was only about one-eighth of a grain. At the same time the subclavian artery was clamped and the vessels divided were all picked up and tied before it was released. This section of the work forms a most valuable addition to our knowledge of what are usually known as local anæsthetics and the whole is arranged and planned in a truly scientific manner.

The War in South Africa, its Cause and Conduct. By A. Conan Doyle. (London: Smith, Elder & Co., and George Newnes, Limited. 1902. Price 6d.)

This little booklet comes very opportunely and serves well to show what were the causes of the war in South Africa and how it has been conducted, and thus to refute in the strongest possible manner many of the wild stories and base calumnies which have been disseminated of late in regard to the methods adopted by our commanders and their troops. The first part of the book is historical, and describes very clearly the various causes which led up to the quarrel, the negotiations, and the endeavours to secure peace which preceded the outbreak of hostilities. Then we come to the war itself, and the way in which it has been managed, and no one can read the latter without recognising on the one hand the immensity of the task and the harassing nature of the work, and on the other hand the courage and humanity with which the task has been performed. In the preface to the book Dr. Conan Doyle says "there never was a war in history in which the right was absolutely on one side, or in which no incidents of the campaign were open to criticism. I do not pretend that it was so here. But I do not think that any unprejudiced man can read the facts without acknowledging that the British Government has done its best to avoid war, and the British Army to wage it with humanity," and this view of the matter is fully borne out in the chapters devoted to the subject. It is certainly a very interesting sixpennyworth, and we hope that Dr. Conan Doyle and his publisher will succeed in their aim, which is to translate it into all European languages, and to send a free copy to every Deputy and every newspaper on the Continent and America.

Home Exercises for Spinal Curvatures, Adapted from Ling's Swedish System of Medical Gymnastics. By Richard Timberg. (London: Simpkin, Marshall, Hamilton, Kent, and Co. Price 2s. net.)

On taking up this book we felt inclined to agree with the opening words of the preface in which the author says that it might with good reason be questioned whether there is any need of, or even any room for, a new treatise on such a subject as spinal curvature. Still, if the book be used as the author says it is intended to be used, we think it may fill a useful place. His aim, it is stated, is to give his book a thoroughly practical form so that the practitioner may safely place it in the hands of his patients to guide them in the performance of those exercises which he may have selected as advisable for the particular case, and which, when possible, he should himself have once or twice superintended. Used in this way we have no doubt that the treatise before us will save the doctor a good deal of trouble, and will give a desirable degree of accuracy to the exercises prescribed. Beginning with a short sketch of the normal condition of the spine and the causes and modes of origin of spinal curvature, the objects to be aimed at by the exercises are explained, after which we have a careful and easily followed description of the exercises, 33 in number. From these various movements selections may be made according to the requirements of each case, and may be prescribed by the appended numbers. It seems to us a useful and unpretending little book.

